# Enhanced Oil Recovery by a Suspension of Core-Shell Polymeric Nanoparticles in Heterogeneous Low-Permeability Oil Reservoirs

**DOI:** 10.3390/nano9040600

**Published:** 2019-04-11

**Authors:** Yunqian Long, Renyi Wang, Baikang Zhu, Xiaohe Huang, Zhe Leng, Liqiao Chen, Fuquan Song

**Affiliations:** 1 Institute of Innovation & Application, Zhejiang Ocean University, Zhoushan 316022, China; lengzhe12345a@163.com (Z.L.); chenlq118@126.com (L.C.); 2 United National-Local Engineering Laboratory of Harbor Oil & Gas Storage and Transportation Technology, Zhejiang Ocean University, Zhoushan 316022, China; zszbk@126.com; 3 School of Petrochemical & Energy Engineering, Zhejiang Ocean University, Zhoushan 316022, China; huangxh@zjou.edu.cn (X.H.); songfuquan@zjou.edu.cn (F.S.)

**Keywords:** core-shell polymeric nanoparticles, enhanced oil recovery, suspension, heterogeneous reservoir, permeability ratio

## Abstract

Polymeric nanoparticle suspension is a newly developed oil-displacing agent for enhanced oil recovery (EOR) in low-permeability reservoirs. In this work, SiO_2_/P(MBAAm-*co*-AM) polymeric nanoparticles were successfully synthesized by a simple distillation–precipitation polymerization method. Due to the introduction of polymer, the SiO_2_/P(MBAAm-*co*-AM) nanoparticles show a favorable swelling performance in aqueous solution, and their particle sizes increase from 631 to 1258 nm as the swelling times increase from 24 to 120 h. The apparent viscosity of SiO_2_/P(MBAAm-*co*-AM) suspension increases with an increase of mass concentration and swelling time, whereas it decreases as the salinity and temperature increase. The SiO_2_/P(MBAAm-*co*-AM) suspension behaves like a non-Newtonian fluid at lower shear rates, yet like a Newtonian fluid at shear rates greater than 300 s^−1^. The EOR tests of the SiO_2_/P(MBAAm-*co*-AM) suspension in heterogeneous, low-permeability cores show that SiO_2_/P(MBAAm-*co*-AM) nanoparticles can effectively improve the sweep efficiency and recover more residual oils. A high permeability ratio can result in a high incremental oil recovery in parallel cores. With an increase of the permeability ratio of parallel cores from 1.40 to 15.49, the ratios of incremental oil recoveries (low permeability/high permeability) change from 7.69/4.61 to 23.61/8.46. This work demonstrates that this SiO_2_/P(MBAAm-*co*-AM) suspension is an excellent conformance control agent for EOR in heterogeneous, low-permeability reservoirs. The findings of this study can help to further the understanding of the mechanisms of EOR using SiO_2_/P(MBAAm-*co*-AM) suspension in heterogeneous, low-permeability reservoirs.

## 1. Introduction

In recent years, due to the severe heterogeneity of reservoir formations, serious water channeling has become a well-recognized problem in the development of low-permeability reservoirs, as the reservoirs enter into the middle or late stages of waterflooding development [1,2,3,4]. When a water flood is carried out in a heterogeneous reservoir, the injected water mainly flows into the high-permeability zones in the formation, leaving most of the low-permeability zones un-swept. This is the so-called conformance problem that is common in heterogeneous reservoirs [5,6,7,8]. Water channeling can lead to high water production, low oil recovery, rapid reaching of the economic limit, and high residual oil saturation in the low-permeability zones of the reservoir formations. Hence, water control is one of the most important goals for the further development of heterogeneous reservoirs [9,10,11,12]. In addition, after many oilfields have experienced water flood for a long period of time, the long-term erosion of the high-permeability zones, caused by water injection, can result in the increased heterogeneity of the reservoirs [13,14,15,16]. As a result, the injected water pours more easily into the producing wells along the high-permeability areas, which makes it more difficult to achieve good water control in the reservoir formations. Therefore, it is crucial to plug the high-permeability zones for the sake of recovering more residual oils from un-swept zones for EOR in heterogeneous reservoirs.

Many technologies have been used to control conformance for improving reservoir heterogeneity, reducing the fluidity of displacement fluid, enlarging the swept volume of displacing fluid, and improving oil recovery in heterogeneous reservoirs. Currently, conformance control technologies have been applied in oilfields, including gel treatment [17], polymer flooding [18,19,20], surfactant-polymer (SP) flooding [21], alkaline-surfactant-polymer (ASP) flooding [22,23], foam flooding [24], and emulsion flooding [25]. Compared with these technologies, it looks promising to use a polymeric nanoparticle suspension as a selective water-shutoff-plugging agent for conformance control treatments in heterogeneous reservoirs. Injecting a large volume of polymeric nanoparticle suspension to plug high-permeability zones and improve oil recovery has become an attractive technology in recent years [26,27,28,29,30]. Polymeric nanoparticle suspension is a system in which polymeric nanoparticles are dispersed in aqueous solution. Polymeric nanoparticles are synthesized by the induction of polymer on the surface of nanoparticles to form a more stable and highly efficient injectant, with a core of nanoparticles and a shell of polymer. There are unparalleled advantages in using polymeric nanoparticle suspension as a conformance control agent [31,32,33,34,35].

A number of polymeric nanoparticle suspensions have been prepared to improve oil recovery in heterogeneous reservoirs in recent years [36,37,38,39]. Lai et al. synthesized modified nano-SiO_2_/AA/AM copolymer (HPMNS) oil displacement agents by free-radical polymerization and carried out indoor displacement tests, and the results indicated that the copolymers can increase both the resistance factor and residual resistance factor in a medium-porous medium under a similar permeability, and they have a stronger mobility control capacity that can effectively improve the EOR [40]. Hu et al. formulated novel, aqueous, partially hydrolyzed, polyacrylamide-based SiO_2_ nanocomposites and found that the inclusion of silica nanoparticles significantly improves the viscosity and viscoelastic properties of partially hydrolyzed polyacrylamide especially under high temperatures and high salinities [41]. Ju et al. used two types of polysilicon nanoparticles in oil fields to improve oil recovery and enhance water injection and confirmed that polysilicon nanoparticles are effective agents for enhancing water injection capability or improving oil recovery [42]. Liu et al. fabricated a novel, star-like, hydrophobically associative polyacrylamide (SHPAM) used in EOR processes in harsh reservoir conditions, and core flooding tests revealed that SHPAM at a concentration of 1500 mg/L increases the oil recovery factor by 20% in sandstone cores [43]. 

It can be seen from the above references that these polymeric nanoparticle suspensions used in high-permeability reservoirs rely on high viscosity to improve the mobility ratio of fluids, which can realize conformance control to improve the EOR. However, they cannot be injected into or move through a porous medium in low-permeability reservoirs. The new direction in polymeric nanoparticle suspension is to develop a suspension of low viscosity, which mainly depends on plugging at pores to realize conformance control. Therefore, the application of polymeric nanoparticle suspensions can be expanded to low-permeability reservoirs. Compared with the polymeric nanoparticle suspensions reported in the literature, the polymeric nanoparticle suspension used in this study has a low bulk viscosity, similar to water, which can assure a good injectivity in low-permeability reservoirs. Simultaneously, the polymeric nanoparticles can swell from their original nano size to micron size in aqueous solution. As the swollen polymeric nanoparticles are injected into the formation, they can plug large pores to reduce the permeability of high-permeability zones, change the flow direction of water, and promote the sweep efficiency of injected water. Moreover, the polymeric nanoparticles can pass through small pores by elastic deformation to achieve deeper seepage in the formation, and they can more effectively reduce the formation permeability away from the water injection well.

**Figure 1:**
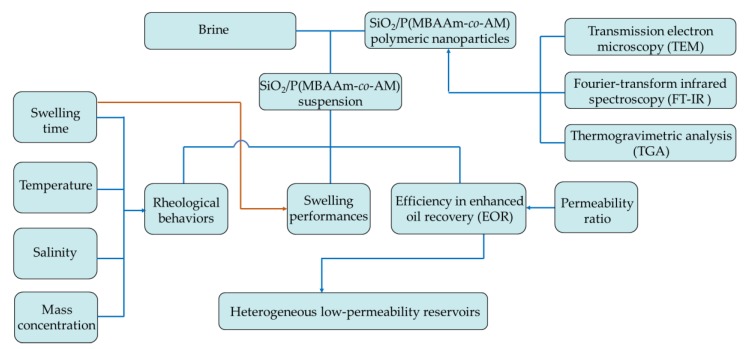
General sketch of this present research.

In this present research, we tried to synthesize SiO_2_/P(MBAAm-*co*-AM) polymeric nanoparticles using a distillation–precipitation polymerization method. The morphology and microstructure of the obtained polymeric nanoparticles were studied via scanning electron microscopy (SEM), transmission electron microscopy (TEM), Fourier-transform infrared spectroscopy (FT-IR), and thermogravimetric analysis (TGA). We investigated the swelling performances of SiO_2_/P(MBAAm-*co*-AM) nanoparticles in aqueous solution using dynamic light scattering (DLS) and the rheological behavior of SiO_2_/P(MBAAm-*co*-AM) suspension using a viscometer. Finally, we investigated the efficiency of SiO_2_/P(MBAAm-*co*-AM) suspension in improving oil recovery in heterogeneous low-permeability reservoirs via parallel cores flooding tests. The general sketch of this present research is shown in Figure 1.

This article is organized as follows. First, materials and methods are presented. Later, the characterizations of SiO_2_ and SiO_2_/P(MBAAm-*co*-AM) nanoparticles are analyzed. Then, the swelling performances of SiO_2_/P(MBAAm-*co*-AM) nanoparticles in an aqueous solution as well as the rheological behaviors and the efficiency of SiO_2_/P(MBAAm-*co*-AM) suspension in EOR are discussed. Finally, the summary and conclusions are presented.

## 2. Materials and Methods

### 2.1. Materials

Tetraethyl orthosilicate (TEOS, 28.5%, Modern Oriental Fine Chemicals Co., Ltd., Beijing, China), acrylamide (AM, Sinopharm Chemical Reagent Co., Ltd., Beijing, China), N, N-methylene-bis-acrylamide (MBAA, Sinopharm Chemical Reagent Co., Ltd., Beijing, China), azodiisobutyronitrile (AIBN, Sinopharm Chemical Reagent Co., Ltd., Beijing, China), acetonitrile (Sinopharm Chemical Reagent Co., Ltd., Beijing, China), ethyl alcohol (Beihua Fine Chemicals Co., Ltd., Beijing, China), and ammonia (25%, Beihua Fine Chemicals Co., Ltd., Beijing, China) were purchased from a local supplier and were reagents of analytical grade. All the solutions were freshly prepared using deionized water. In addition, a crude oil sample with a density of 0.895 g/cm^3^ and a viscosity of 15.2 mPa·s (45 °C), and eight cores with lengths of 5.0 cm and diameters of 2.5 cm were obtained from a Daqing oil reservoir for the displacement experiments. The synthetic formation water used for the displacement experiments was a brine with a total dissolved solids (TDS) value of 5380 mg/L.

### 2.2. Synthesis of SiO_2_ Nanoparticles

SiO_2_ nanoparticles were synthesized by a Stӧber process [44]. Briefly, TEOS (11.2 mL), ethyl alcohol (84.1 mL), and deionized water (17.1 mL) were added into a 250 mL conical flask. After that, ammonia (4.76 mL) was poured into the above solution under continuous magnetic stirring. After the solution was allowed to react for 6 h under magnetic stirring, the SiO2 nanoparticles were obtained by being centrifuged, washed with ethanol, and dried at 70 °C for 8 h in a vacuum.

### 2.3. Synthesis of SiO_2_/P(MBAAm-co-AM) Polymeric Nanoparticles

SiO_2_/P(MBAAm-*co*-AM) polymeric nanoparticles were synthesized by a distillation– precipitation polymerization method [45]. Briefly, the as-prepared SiO_2_ nanoparticles (0.5 g) were suspended in acetonitrile (40 mL) in a 250 mL three-neck flask connected to a reflux condenser. MBAA (0.2 g) and AM (0.5 g) were dissolved in the above suspension and sonicated for 10 min at room temperature. Then, AIBN (0.1 g) was added into the above system and the mixture was sonicated for 5 min. After that, the above mixture was reacted at 90 °C for 15 min and then distilled at 115 °C for 90 min with a reflux ratio of 2 until all solvents were distilled out. The products were dissolved in ethyl alcohol and sonicated for 10 min. Finally, SiO_2_/P(MBAAm-*co*-AM) polymeric nanoparticles were obtained after being centrifuged, washed with deionized water, and dried at 50 °C for 12 h in a vacuum.

### 2.4. Characterization

Scanning electron microscopy (SEM, S-4500 Hitachi, Tokyo, Japan) and transmission electron microscopy (TEM, JEM-200CX JEOL, Tokyo, Japan) were employed to characterize the morphology of the samples. The functional groups of the products were characterized via Fourier-transform infrared spectroscopy (FT-IR, Nexus 670 Thermo Nicolet, Wisconsin, USA). The thermogravimetric analysis (TGA) of the products was performed on a thermogravimetric analyzer (SDT Q600 TA, New Castle, DE, USA) at a heating rate of 10 °C/min in nitrogen.

### 2.5. Particle Size Distribution Measurements

SiO_2_/P(MBAAm-*co*-AM) polymeric nanoparticles can mixed with water to form a suspension. In suspension, these polymeric nanoparticles can absorb water, and swell with their diameters increasing from their original nano size or submicron size to micron size. Thus, particle size distribution measurements were carried out by dynamic light scattering (DLS), using a laser particle size analyzer (Mastersizer 2000 Malvern, Worcestershire, UK). The particle size distribution curves of SiO_2_/P(MBAAm-*co*-AM) polymeric nanoparticles were determined at various swelling times in suspension with a salinity of 5.0 g/L at 40 °C. Medium diameters of polymeric nanoparticles at various swelling times were obtained by analyzing the particle size distribution curves. Then, the swelling property was quantificationally described by the swelling ratio, defined as follows:

(1) $$Q=\frac{D_{2}-D_{1}}{D_{1}}$$

where *Q* is the swelling ratio, *D*_2_ is the medium diameter of swollen polymeric nanoparticles at each time interval (μm), and *D*_1_ is the average particle diameter of initial dry composite nanoparticles (μm).

### 2.6. Viscosity Measurements

The viscosity measurements were carried out using a viscometer (LV-DV2T Brookfield, Middleboro, MA, USA). The apparent viscosities of the SiO_2_/P(MBAAm-*co*-AM) suspensions were measured at a shear rate of 7.34 s^−1^ to investigate the effect of mass concentration, temperature, salinity, and swelling time. The apparent viscosities of the SiO_2_/P(MBAAm-*co*-AM) suspensions versus shear rates were determined at mass concentrations of 0.05, 0.5, and 1.5 g/L, temperatures of 40, 60, and 80 °C, salinities of 2, 5, and 10 g/L, and swelling times of 24, 120, and 240 h.

### 2.7. Oil Displacement Experiments

**Figure 2:**
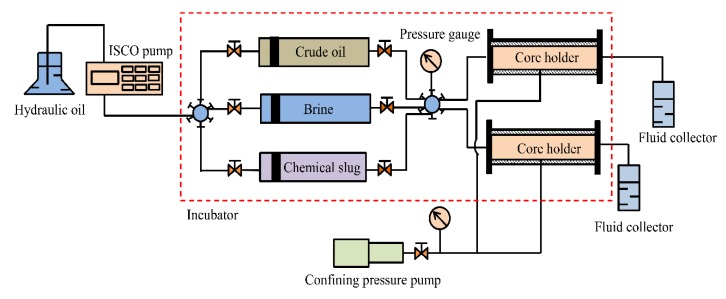
Schematic of the experimental set-up. The experimental set-up consisted of two hydrostatic core holders, a pump to maintain confining pressure, a displacement pump for the flooding agent, and two fluid accumulators for holding the collected oil sample, brine, and chemical slug.

Before starting the flooding experiments, cores were properly cleaned and dried at 150 °C for 72 h. Their porosity was measured using the weight method, and permeability measurements were conducted using Darcy’s law. In each flood test, the core was first saturated with brine, then mounted in hydrostatic core holders at a confining pressure of 3 MPa; finally, the samples were flooded with crude oil at a constant injection rate of 0.2 mL/min until the condition of irreducible water saturation was achieved in core. The flood experiments were carried out at 45 °C. The core was aged for 48 h to achieve the wettability state at 45 °C. After that, the core sample was flooded with brine at a flow rate of 0.2 mL/min until the water cut was about 98%. Subsequently, a chemical slug of suspension prepared by mixing 1.5 g SiO_2_/P(MBAAm-*co*-AM) polymeric nanoparticles with 1 L of water was injected at a constant injection rate of 0.2 mL/min, followed by an extended waterflood at the same flow rate until the oil production stopped. The recovered crude oil and brine were collected in fluid collectors, and the injection pressure data were recorded. The experimental set-up used for the core-flooding experiments is shown in Figure 2.

## 3. Results and Discussion

### 3.1. Characterization of SiO_2_/P(MBAAm-co-AM) Nanoparticles

**Figure 3:**
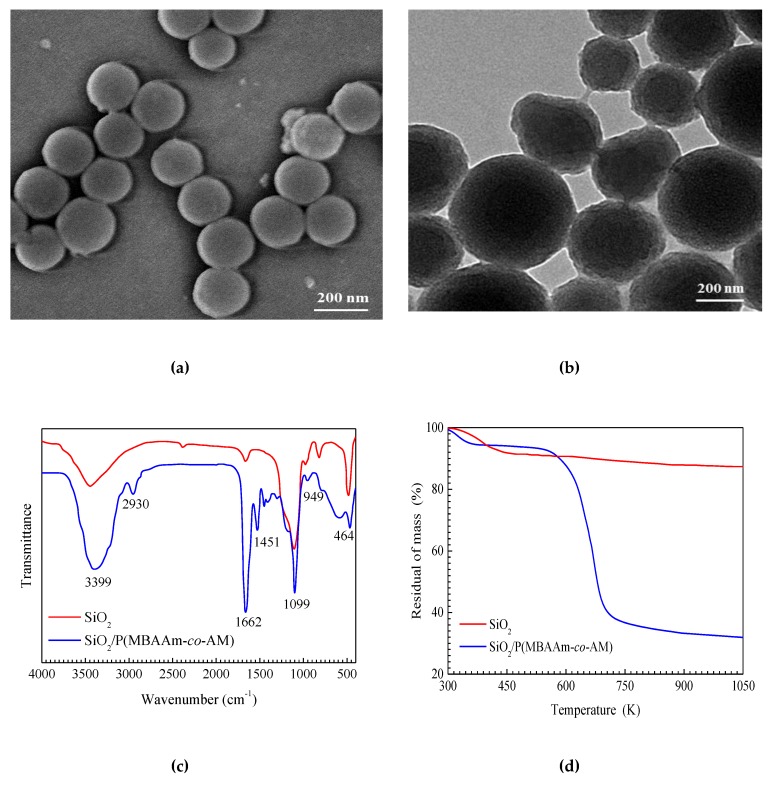
SEM image of SiO_2_ nanoparticles (**a**); TEM image of SiO_2_/P(MBAAm-*co*-AM) nanoparticles (**b**); and FT-IR spectra (**c**) and TGA curves (**d**) of SiO_2_, SiO_2_/P(MBAAm-*co*-AM).

The size, shape, and surface morphologies of SiO_2_ and SiO_2_/P(MBAAm-*co*-AM) nanoparticles were characterized by SEM and TEM, as shown in Figure 3. It was found from Figure 3a that SiO_2_ nanoparticles are spherical and uniform, and are approximately 200 nm in diameter. The TEM image (Figure 3b) indicates that SiO_2_/P(MBAAm-*co*-AM) nanoparticles still retain their spherical morphologies, and consist of a SiO_2_ core and a shell of polymer, with diameters ranging from 200 to 400 nm. The thickness of the polymer layer is in the range of 50–100 nm. Thus, it can be considered that SiO_2_/P(MBAAm-*co*-AM) nanoparticles were successfully synthesized.

Figure 3c shows the FT-IR spectra of SiO_2_ and SiO_2_/P(MBAAm-*co*-AM). The peaks at 3415, 1099, 957, 806, and 464 cm^−1^ are the characteristic peaks of SiO_2_ [46]. The peaks at 3732 and 1641 cm^−1^ may be due to the vibration of water molecules [47]. As seen in the spectrum of SiO_2_/P(MBAAm-*co*-AM), the peaks at 1099 and 949 cm^−1^ are assigned as the characteristic peaks of SiO_2_, and associated with the symmetric stretching vibration of the Si–O–Si bond and the stretching vibration of the Si–OH bond, respectively [48]. The peaks at 3399 and 1662 cm^−1^ are attributed to the stretching vibration of the N–H bond and the C=O bond in the amide group, respectively [49]. The peaks at 2930 and 1451 cm^−1^ are ascribed to the stretching vibration of the C–H bond and the bending vibration of the C–H bond in the saturated alkyl group, respectively [50]. Therefore, this spectrum demonstrates the successful preparation of SiO_2_/P(MBAAm-*co*-AM) nanoparticles as well.

Figure 3d shows the TGA curves of SiO_2_ and SiO_2_/P(MBAAm-*co*-AM). The weight losses of SiO_2_ and SiO_2_/P(MBAAm-*co*-AM) prior to reaching 458 K are attributed to the loss of adsorbed water [51]. SiO_2_ shows a small degradation (a loss of 4.26%) between 458 and 1050 K. The TGA curve of SiO_2_/P(MBAAm-*co*-AM) shows three steps. The first step occurs at 300–458 K due to a 6.02% loss, which is attributed to the loss of physisorbed water. The second and third steps are at 458–737 K and at 737–1050 K, with losses of 56.67% and 6.52%, respectively. These are ascribed to the loss from polymer pyrolysis and dehydroxylation from SiO_2_, respectively [52]. According to the total loss of SiO_2_/P(MBAAm-*co*-AM), about 56.67% of P(MBAAm-*co*-AM) was grafted on the surface of SiO_2_, and the residue is about 30.79% of SiO_2_. These results further demonstrate the successful preparation of P(MBAAm-*co*-AM) grafted on the surface of SiO_2_.

### 3.2. Swelling Performances of SiO_2_/P(MBAAm-co-AM) Nanoparticles in a Suspension

**Figure 4:**
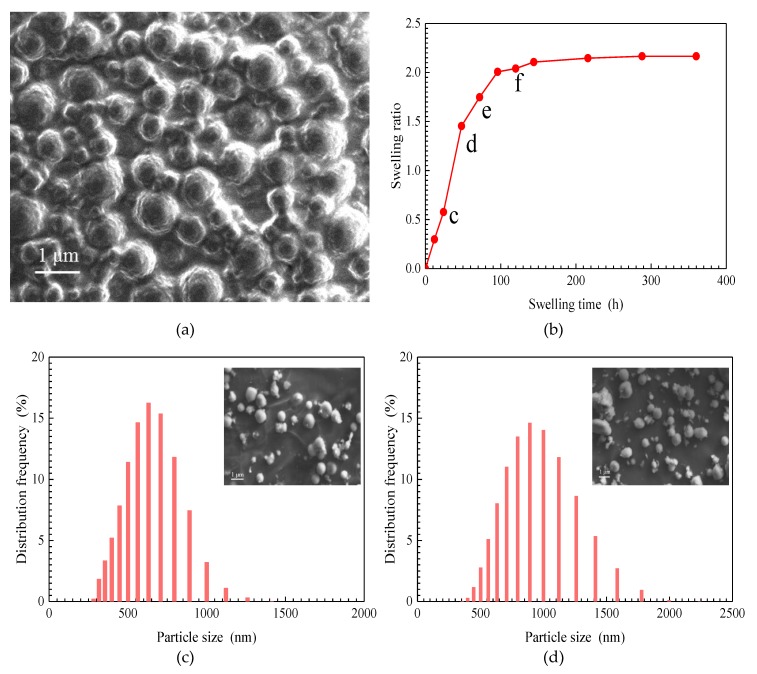

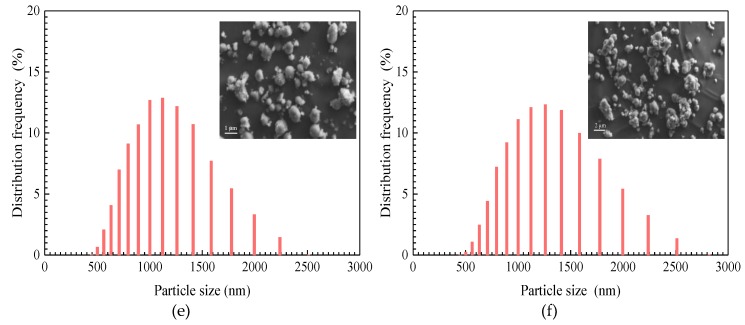
SEM image of SiO_2_/P(MBAAm-*co*-AM) suspension (**a**); the effect of swelling time on swelling ratio of SiO_2_/P(MBAAm-*co*-AM) nanoparticles (**b**, total dissolved solids (TDS) = 5 g/L, T = 40 °C); and particle size distribution curves of SiO_2_/P(MBAAm-*co*-AM) nanoparticles in suspension at swelling times of 24 h (**c**), 48 h (**d**), 72 h (**e**), and 120 h (**f**). Insets are the SEM images of SiO_2_/P(MBAAm-*co*-AM) suspension.

An SEM image of a SiO_2_/P(MBAAm-*co*-AM) suspension swollen for 24 h at 5000 mg/L is shown in Figure 4a. In this suspension, the size of the SiO_2_/P(MBAAm-*co*-AM) nanoparticles is obviously larger than that of their dry samples. The size of a single SiO_2_/P(MBAAm-*co*-AM) nanoparticle is between 250 and 1000 nm, and some nanoparticles are agglomerated in the high concentration of the SiO_2_/P(MBAAm-*co*-AM) suspension. The agglomerated nanoparticles range in size from 800 to 1500 nm. This result proves that SiO_2_/P(MBAAm-*co*-AM) nanoparticles can absorb water to swell in aqueous solution. In addition, a Mastersizer 2000 laser particle size analyzer was used in this study to provide more details about SiO_2_/P(MBAAm-*co*-AM) nanoparticles in a suspension.

The plot of the swelling ratio of the SiO_2_/P(MBAAm-*co*-AM) nanoparticles versus swelling time, at 1500 mg/L, is shown in Figure 4b. The swelling ratio of SiO_2_/P(MBAAm-*co*-AM) nanoparticles swiftly increases at the initial stage and then slows until the swelling reaches equilibrium. Under this experimental condition, the equilibrium swelling ratio of SiO_2_/P(MBAAm-*co*-AM) nanoparticles can reach up to about 2.

The particle size distribution curves of SiO_2_/P(MBAAm-*co*-AM) nanoparticles at swelling times of 24, 48, 72, and 120 h were determined, as shown in Figure 4c–f. It was found that the particle size distribution curves approximately match the normal distribution. A shift of particle size distributions to larger sizes occurs with an increase of swelling time, with a concomitant decrease of the height of the predominant peak in volume distribution. As swelling time increases from 24 to 120 h, the particle size with the largest distribution frequency increases from 631 to 1258 nm, with a decrease of distribution frequency from 16.24 to 12.32%. SEM images of SiO_2_/P(MBAAm-*co*-AM) suspension, shown as the insets in Figure 4c–f, qualitatively reflect both the swelling process and change regulation of SiO_2_/P(MBAAm-*co*-AM) nanoparticles in water.

### 3.3. Rheological Behaviors of SiO_2_/P(MBAAm-co-AM) Suspension

**Figure 5:**
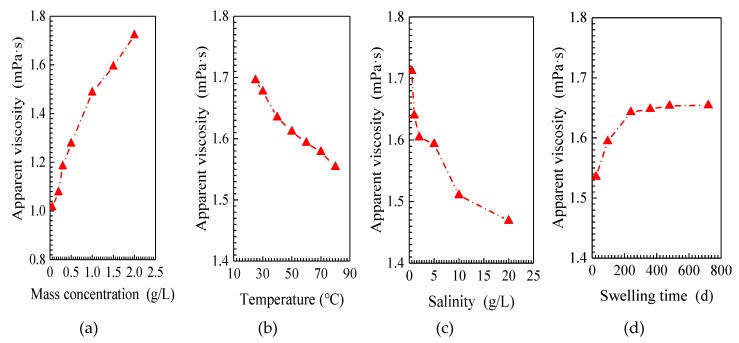
Effect of mass concentration (**a**, T = 60 °C, TDS = 5 g/L, and t = 120 h), temperature (**b**, C = 1.5 g/L, TDS = 5 g/L, and t = 120 h), salinity (**c**, T = 60 °C, C = 1.5 g/L, and t = 120 h), and swelling time (**d**, T = 60 °C, C = 1.5 g/L, and TDS = 5 g/L) on viscosity properties of SiO_2_/P(MBAAm-*co*-AM) suspension.

The apparent viscosity of the SiO_2_/P(MBAAm-*co*-AM) suspension versus mass concentration of SiO_2_/P(MBAAm-*co*-AM) nanoparticles is shown in Figure 5a. As shown, at a higher mass concentration, the SiO_2_/P(MBAAm-*co*-AM) suspension exhibits a larger apparent viscosity. This is because with increasing mass concentration, the number of SiO_2_/P(MBAAm-*co*-AM) nanoparticle collisions with water molecules increases, which results in an increase of apparent viscosity [53].

The apparent viscosity of the SiO_2_/P(MBAAm-*co*-AM) suspension versus temperature is shown in Figure 5b. According to Figure 5b, the apparent viscosity of the SiO_2_/P(MBAAm-*co*-AM) suspension decreases with increasing temperature. As the temperature rises, it reduces the intermolecular force, so the SiO_2_/P(MBAAm-*co*-AM) suspension moves more easily, leading to a decrease of apparent viscosity.

Figure 5c shows the apparent viscosity of the SiO_2_/P(MBAAm-*co*-AM) suspension as a function of the salinity. As shown in Figure 5c, the apparent viscosity of the SiO_2_/P(MBAAm-*co*-AM) suspension decreases with an increase of salinity. This means that with an increase of salinity, the particle size of the swollen nanoparticles decreases to reduce the number of nanoparticles collisions with water molecules, resulting in a decrease of apparent viscosity [54].

Figure 5d shows the apparent viscosity of the SiO_2_/P(MBAAm-*co*-AM) suspension as a function of the swelling time. The apparent viscosity of the SiO_2_/P(MBAAm-*co*-AM) suspension rapidly increases at the initial stage and then slowly increases until the apparent viscosity remains constant at swelling times greater than 200 h. With an increase of swelling time, SiO_2_/P(MBAAm-*co*-AM) nanoparticles swell to enlarge their particle sizes, so the number of nanoparticles that collide with water molecules increases, resulting in an increase of apparent viscosity. However, when the swelling reaches equilibrium, the particle size does not enlarge, and the number of collisions with water molecules does not increase, so the apparent viscosity remains constant.

**Figure 6:**
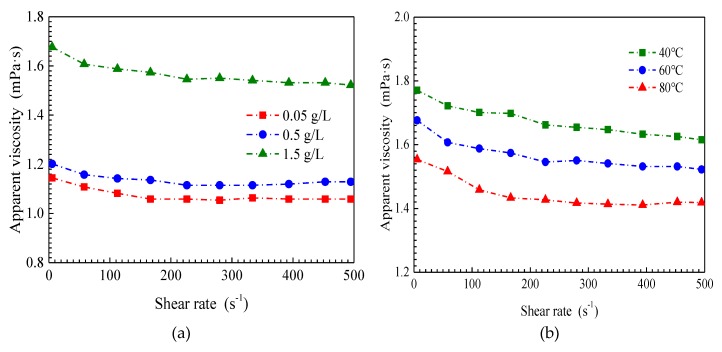

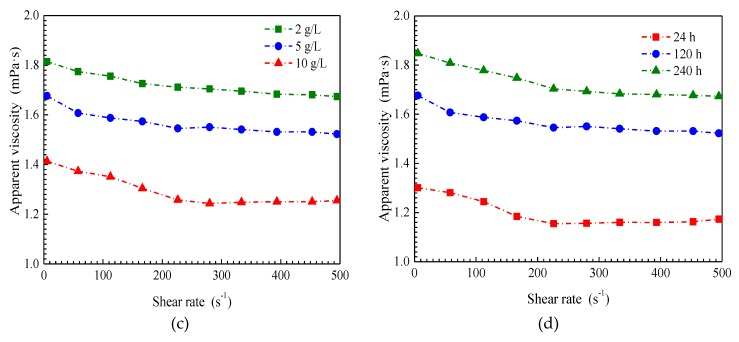
Effect of mass concentration (**a**, T = 60 °C, TDS = 5 g/L, and t = 120 h), temperature (**b**, C = 1.5 g/L, TDS = 5 g/L, and t = 120 h), salinity (**c**, T = 60 °C, C = 1.5 g/L, and t = 120 h), and swelling time (**d**, T = 60 °C, C = 1.5 g/L, and TDS = 5 g/L) on rheological properties of SiO_2_/P(MBAAm-*co*-AM) suspension.

The apparent viscosities of the SiO_2_/P(MBAAm-*co*-AM) suspension with respect to shear rate are presented in Figure 6 at various mass concentrations of SiO_2_/P(MBAAm-*co*-AM) nanoparticles, temperatures, salinities, and swelling times. Under all experimental conditions, the apparent viscosity of the SiO_2_/P(MBAAm-*co*-AM) suspension versus shear rate follows a similar trend. At lower shear rates, the apparent viscosity of the SiO_2_/P(MBAAm-*co*-AM) suspension decreases with an increase of shear rate, and we can consider the behavior of the SiO_2_/P(MBAAm-*co*-AM) suspension as non-Newtonian. At shear rates greater than 300 s^−1^, the constant apparent viscosity of the SiO_2_/P(MBAAm-*co*-AM) suspension after changing shear rate indicates that the apparent viscosity does not depend on shear rate and represents Newtonian fluid behavior.

### 3.4. EOR by a SiO_2_/P(MBAAm-co-AM) Suspension in Parallel Cores

SiO_2_/P(MBAAm-*co*-AM) composite nanoparticles were mixed with water to form a SiO_2_/P(MBAAm-*co*-AM) suspension. The concentration of SiO_2_/P(MBAAm-*co*-AM) composite nanoparticles was 1500 mg/L. Parallel cores flooding tests were carried out to investigate the efficiency of SiO_2_/P(MBAAm-*co*-AM) suspension in EOR in heterogeneous low-permeability reservoirs. Core parameters and test results are shown in Table 1. In four tests, the slug size of a SiO_2_/P(MBAAm-*co*-AM) suspension was 0.5 PV (pore volume) and the permeability ratio of parallel cores ranged from 1.40 to 15.49.

#### 3.4.1. Effect of Permeability Ratio on Fractional Flows

**Figure 7:**
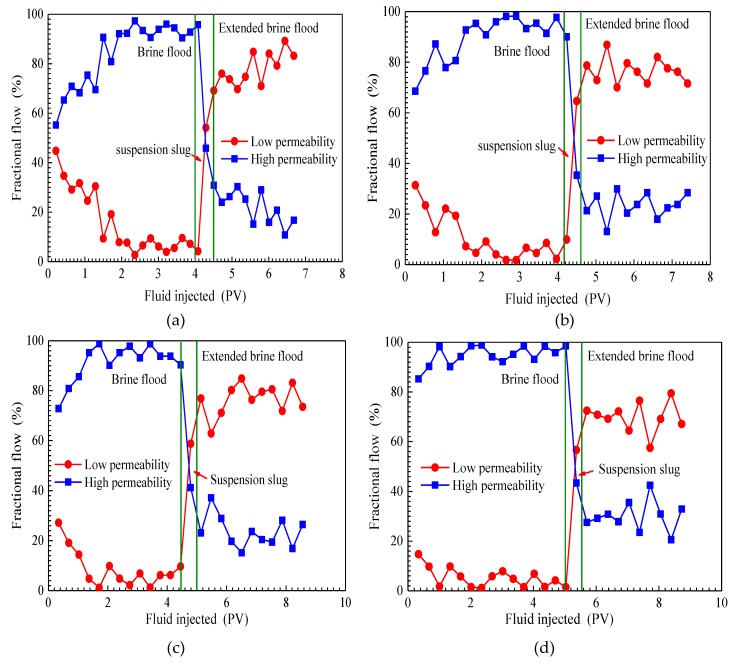
Fractional flow curves of four parallel cores flooding tests. Permeability ratio: (**a**) 1.40; (**b**) 4.36; (**c**) 8.28; (**d**) 15.49.

The fractional flow curves of three flooding stages, which included an initial brine flood, a SiO_2_/P(MBAAm-*co*-AM) suspension slug injection, and an extended brine flood in four parallel cores tests, are plotted in Figure 7. As shown in Figure 7, the same changes of fractional flow curves are observed in four flooding tests of different permeability ratios. In the initial brine flood stage, the fractional flows in high-permeability cores increase with injection, while the fractional flows in low-permeability cores decrease with injection. Moreover, the difference of fractional flows in high- and low-permeability cores increase as brines are injected. After brine breaks through in high-permeability cores, brine flooding mainly passes through high-permeability cores. Therefore, at the end of brine flooding, the fractional flows in high-permeability cores are greater than 90%, and in low-permeability cores, less than 10%. As SiO_2_/P(MBAAm-*co*-AM) suspension slug is injected, the fractional flows in high-permeability cores sharply decrease and in those low-permeability cores rapidly increase. Then, the fractional flows in low-permeability cores exceed those in high-permeability cores. At the end of SiO_2_/P(MBAAm-*co*-AM) suspension slug injection, the fractional flows increase to more than 60% in low-permeability cores, and decrease to less than 40% in high-permeability cores. The above changes of fractional flows in high- and low-permeability cores confirm that SiO_2_/P(MBAAm-*co*-AM) nanoparticles can block water channels in high-permeability cores to produce fluid diversion and enlarge the swept volume in low-permeability cores [55]. As the extended brine flooding starts, the fractional flows in high- and low-permeability cores both fluctuate with the migration of SiO_2_/P(MBAAm-*co*-AM) nanoparticles [56]. The fractional flows in low-permeability cores are always higher than those of high-permeability cores throughout the extended brine flood. This indicates that most of the SiO_2_/P(MBAAm-*co*-AM) nanoparticles flow into high-permeability cores and increase the flow resistance of the following brine.

In addition, it can be seen from Figure 7 that the permeability ratio of parallel cores has a significant effect on fractional flows. During the brine flooding, with an increase of permeability ratio from 1.40 to 15.49, the initial difference of fractional flows in two cores increases from 10 to 60%, and the breakthrough time of brine in high-permeability core decreases from 2.0 PV to 1.0 PV. Thus, it is evident that brine flooding is less effective as the permeability ratio of two cores becomes larger. When extended brine flood finishes, the difference of fractional flows in parallel cores decreases from 70 to 40% as the permeability ratio increases from 1.40 to 15.49. The above results indicate that the greater the permeability ratio of two cores is, the more effective the profile control of the SiO_2_/P(MBAAm-*co*-AM) nanoparticles. When SiO_2_/P(MBAAm-*co*-AM) nanoparticles enter into cores, they can generate plugging at a pore throat, and some large nanoparticles are detained in pores. However, some nanoparticles can pass through pore throats by deformation under a driving pressure due to their ability to be elastic; then, the deformed nanoparticles can recover to their original shapes after entering into a larger pore. In addition, some nanoparticles break into smaller particles at the pore throat, which can pass through smaller pore throats. The plugging of these SiO_2_/P(MBAAm-*co*-AM) nanoparticles increases the flow resistance of blocked regions and diverts the fluid into low-resistance regions. Therefore, SiO_2_/P(MBAAm-*co*-AM) nanoparticles can effectively reduce the heterogeneity of reservoirs and improve the sweep efficiency.

#### 3.4.2. Effect of Permeability Ratio on Pressure Drop

**Figure 8:**
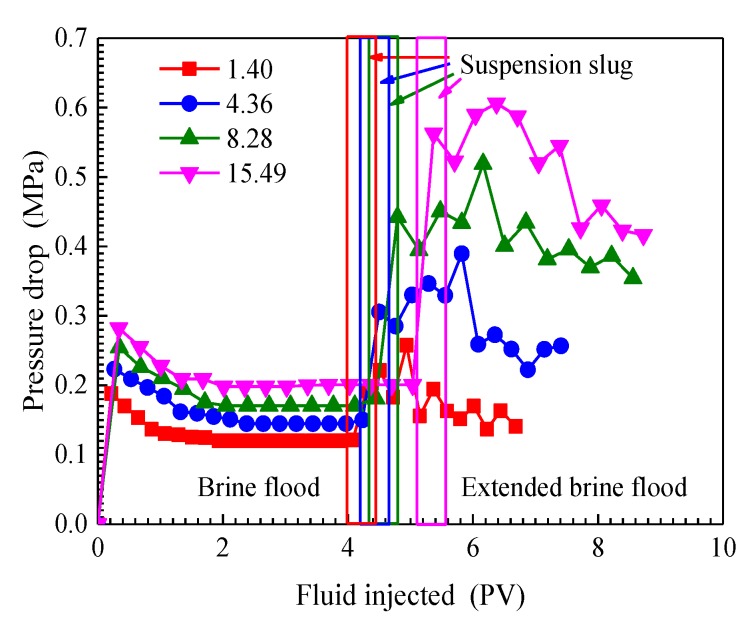
Curves of pressure drop as a function of the pore volume of fluid injected under different permeability ratios.

Pressure drop is defined as the pressure difference between the core inlet and outlet, which was determined by using pressure gauge [5]. The curves of pressure drop as a function of the pore volume of fluid injected under different permeability ratios are plotted in Figure 8. Each curve in Figure 8 reflects the similar variation of pressure drop with the pore volume of fluid injected. During the brine flooding, the pressure drop sharply rises in a very short time, and then falls. When brine breaks through into a high-permeability core, the pressure drop starts to remain almost constant. When the SiO_2_/P(MBAAm-*co*-AM) suspension slug injection starts, pressure drop raises rapidly, with tiny fluctuations. In the extended brine flood stage, the fluctuated pressure drop continues to rise until it reaches a maximum and then declines. These results indicate that the behaviors of SiO_2_/P(MBAAm-*co*-AM) nanoparticles at pore throats can result in a fluctuation of pressure drop. According to Darcy’s law, the fluctuated pressure drop can lead to a fluctuation of fractional flow, which is consistent with the experimental results of fractional flow. In addition, pressure drop in different parallel cores increases with increasing permeability ratios. During the extended brine flood, the maximum pressure drop rises from 0.26 MPa to 0.61 MPa when the permeability ratio increases from 1.40 to 15.49. As the permeability ratio of the parallel cores increases, the blockage of SiO_2_/P(MBAAm-*co*-AM) nanoparticles in the core becomes stronger so that a greater pressure drop is obtained. The fluid can enter into smaller pores, and its swept volume is enlarged. Hence, it can be concluded that SiO_2_/P(MBAAm-*co*-AM) nanoparticles have a better profile control effect for more heterogeneous media.

#### 3.4.3. Effect of Permeability Ratio on EOR

**Figure 9:**
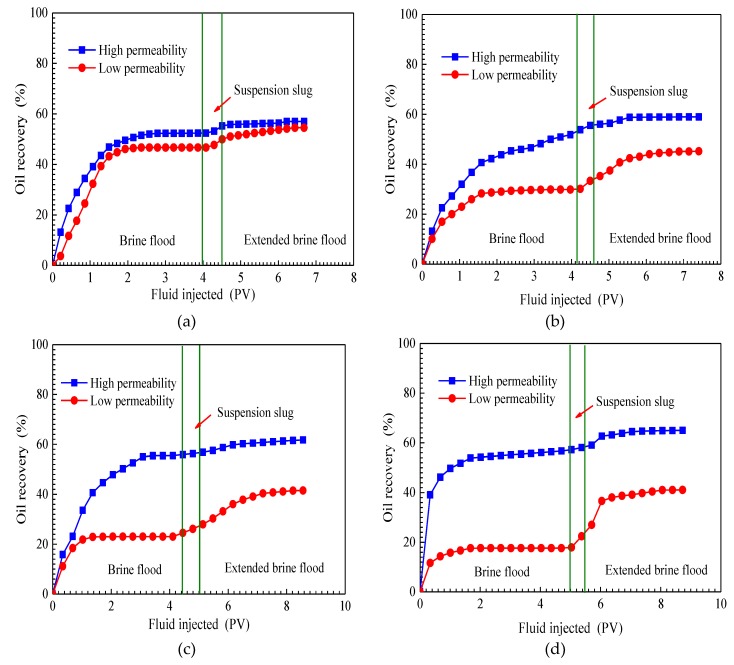
Cumulative oil recoveries of four parallel cores flooding tests. Permeability ratio: (**a**) 1.40; (**b**) 4.36; (**c**) 8.28; (**d**) 15.49.

The cumulative oil recoveries of four parallel core flooding tests are plotted as a function of the pore volume of fluid injected in Figure 9. Each of the diagrams in Figure 9 has two curves representing a high-permeability core and low-permeability core. The cumulative oil recovery includes all three flooding stages—initial brine flood, SiO_2_/P(MBAAm-*co*-AM) suspension slug injection, and extended brine flood. The results of four tests show that during the initial brine flooding, oil recoveries of low-permeability cores are less than those of high-permeability cores. Moreover, this difference increases with an increasing permeability ratio of parallel cores. When permeability ratios are 1.40, 4.36, 8.28, and 15.49, brine flood recovers 52.37%, 53.84%, 55.49%, and 56.69% of the initial oil in place (IOIP) of high-permeability cores, respectively, which are all higher than the cumulative oil recoveries of low-permeability cores in the same parallel cores: 46.70%, 29.79%, 22.99%, and 17.61% IOIP. The test results illustrate that a high permeability ratio can result in high residual oil saturation in low-permeability cores.

For the purpose of improving oil recovery in heterogeneous reservoirs, a slug of SiO_2_/P(MBAAm-*co*-AM) suspension was injected into parallel cores. As seen in Figure 9, in the slug injection stage, both oil recovery curves rise, and the curve of the high-permeability core rises much slower than that of the low-permeability core. This indicates that most SiO_2_/P(MBAAm-*co*-AM) composite nanoparticles enter into high-permeability cores to perform effective plugging at throats. Therefore, the flow resistance in high-permeability cores is increased, and the major flow passage in parallel cores can be diverted from the high-permeability core to the low-permeability core by the injected SiO_2_/P(MBAAm-*co*-AM) suspension. Because most of the injected SiO_2_/P(MBAAm-*co*-AM) composite nanoparticles are detained in high-permeability cores, the effect of enlarging the sweep volume continues during the extended brine flood. It can be seen from the oil recovery curves of the extended brine flood that the cumulative oil recoveries in low-permeability cores are 54.39%, 46.06%, 41.57%, and 41.22% IOIP for tests 1, 2, 3, and 4, respectively, which are still less than those of the high-permeability cores in the same parallel cores: 56.98%, 58.82%, 61.74%, and 65.15% IOIP for tests 1, 2, 3, and 4. However, compared with the test results of brine flood, the difference between the oil recovery of a high-permeability core and that of a low-permeability core in the same parallel cores reduces after the extended brine flood. Meanwhile, it can be noticed from Table 1 that the incremental oil recoveries in low-permeability cores, by SiO_2_/P(MBAAm-*co*-AM) suspension injection and the following extended brine flood, are much higher than those in high-permeability cores. The ratios of incremental oil recoveries (low permeability/high permeability) are 7.69/4.61, 16.27/4.98, 18.58/6.25, and 23.61/8.46 for tests 1, 2, 3, and 4, respectively. Thus, it is obvious that a high permeability ratio can result in a high incremental oil recovery in a low-permeability core. The above results illustrate that the injection of SiO_2_/P(MBAAm-*co*-AM) suspension can effectively improve the sweep efficiency and recover more residual oil in a heterogeneous, low-permeability reservoir. Moreover, the effect of SiO_2_/P(MBAAm-*co*-AM) suspension on enlarging the sweep efficiency becomes more noticeable in a more heterogeneous, low-permeability reservoir.

**Figure 10:**
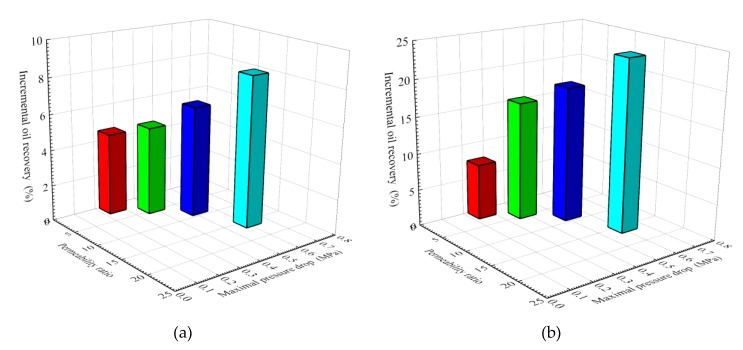
Effect of permeability ratio and maximal pressure drop on incremental oil recovery: (**a**) high-permeability cores; (**b**) low-permeability cores.

In order to investigate the relationship between pressure drop and EOR in four parallel core flooding tests, the incremental oil recoveries in high- and low-permeability cores were plotted as a function of the maximal pressure drop at each permeability ratio, as shown in Figure 10. The largest maximum pressure drop occurs at a higher permeability ratio. It can be observed that, with an increase of permeability ratio and maximal pressure drop, an improvement in EOR is denoted by an increase from 4.61 to 8.46% in high-permeability cores (Figure 10a) and from 7.69 to 23.61% in low-permeability cores (Figure 10b). This indicates that the SiO_2_/P(MBAAm-*co*-AM) suspension is appropriate to be applied to improve oil recovery in heterogeneous, low-permeability reservoirs.

## 4. Conclusions

In summary, SiO_2_/P(MBAAm-*co*-AM) composite nanoparticles have been successfully synthesized by a distillation–precipitation polymerization method. Their morphology and microstructure were ascertained by TEM, FT-IR, and TGA. The swelling performances of SiO_2_/P(MBAAm-*co*-AM) nanoparticles and the rheological behaviors and efficiency of SiO_2_/P(MBAAm-*co*-AM) suspension in EOR were investigated. The main results can be summarized as follows:

(1) SiO_2_/P(MBAAm-*co*-AM) nanoparticles exhibit a favorable swelling property in an aqueous solution and their particle sizes increase from 631 to 1258 nm as the swelling times increase from 24 to 120 h.

(2) The apparent viscosity of SiO_2_/P(MBAAm-*co*-AM) suspension increases with an increase of mass concentration and swelling time, whereas it decreases as salinity and temperature increase. The SiO_2_/P(MBAAm-*co*-AM) suspension behaves like a non-Newtonian fluid at lower shear rates, whereas it represents a Newtonian fluid behavior at shear rates greater than 300 s^−1^.

(3) SiO_2_/P(MBAAm-*co*-AM) nanoparticles can effectively reduce the heterogeneity of reservoirs and improve the sweep efficiency. A high permeability ratio can result in a high incremental oil recovery in parallel cores. When the permeability ratio increases from 1.40 to 15.49, the incremental oil recoveries rise from 4.61 to 8.46% and from 7.69 to 23.61% in high- and low-permeability cores, respectively.

Despite the fact that the SiO_2_/P(MBAAm-*co*-AM) suspension is appropriate to be applied to improve oil recovery in heterogeneous, low-permeability reservoirs, there are two major limitations in this study that could be addressed in future research. First, this study focused on the effect of permeability ratio on EOR, yet it did not discuss the influence of nanoparticle properties on EOR. Second, this study lacked core samples from representative blocks of low-permeability reservoirs.

## Figures and Tables

**Table 1:** Efficiency of enhanced oil recovery (EOR) in heterogeneous parallel cores.

Test	Permeability (10^−3^ μm^2^)	Permeability Ratio	Recovery of Brine Flood (%)	Final Oil Recovery (%)	Incremental Oil Recovery (%)
1	High 20.28	1.40	52.37	56.98	4.61
	Low 14.47		46.70	54.39	7.69
2	High 75.82	4.36	53.84	58.82	4.98
	Low 17.39		29.79	46.06	16.27
3	High 66.45	8.28	55.49	61.74	6.25
	Low 8.03		22.99	41.57	18.58
4	High 64.74	15.49	56.69	65.15	8.46
	Low 4.18		17.61	41.22	23.61
